# The economic impact of a local, collaborative, stepped, and personalized care management for older people with chronic diseases: results from the randomized comparative effectiveness LoChro-trial

**DOI:** 10.1186/s12913-023-10401-1

**Published:** 2023-12-15

**Authors:** Klaus Kaier, Gloria Metzner, Lukas Horstmeier, Eva Maria Bitzer, Bernhard Heimbach, Jasmin Kiekert, Sebastian Voigt-Radloff, Erik Farin-Glattacker

**Affiliations:** 1 Institute of Medical Biometry and Statistics, Faculty of Medicine and Medical Center, University of Freiburg, Hugstetter Str. 49, 79106 Freiburg, Germany; 2 Section of Health Care Research and Rehabilitation Research, Institute of Medical Biometry and Statistics, Faculty of Medicine and Medical Center, University of Freiburg, Freiburg, Germany; 3 University of Education Freiburg, Freiburg, Germany; 4 Centre for Geriatric Medicine and Gerontology, Faculty of Medicine and Medical Center, University of Freiburg, Freiburg, Germany; 5 Catholic University of Applied Sciences Freiburg, Karlstraße 63, 79104 Freiburg, Germany

**Keywords:** RCT, Cost-effectiveness, Economic evaluation, Cost of care, Integrated care, Multimorbidity

## Abstract

**Background:**

Within the ageing population of Western societies, an increasing number of older people have multiple chronic conditions. Because multiple health problems require the involvement of several health professionals, multimorbid older people often face a fragmented health care system. To address these challenges, in a two-group parallel randomized controlled trial, a newly developed care management approach (LoChro-Care) was compared with usual care.

**Methods:**

LoChro-Care consists of individualized care provided by chronic care managers with 7 to 16 contacts over 12 months. Patients aged 65 + with chronic conditions were recruited from inpatient and outpatient departments. Healthcare utilization costs are calculated by using an adapted version of the generic, self-reporting FIMA©-questionnaire with the application of standardized unit costs. Questionnaires were given at 3 time points (T_0_ baseline, T_1_ after 12 months, T_2_ after 18 months). The primary outcome was overall 3-month costs of healthcare utilization at T_1_ and T_2_. The data were analyzed using generalized linear models with log-link and gamma distribution and adjustment for age, sex, level of care as well as the 3-month costs of care at T_0_.

**Results:**

Three hundred thirty patients were analyzed. The results showed no significant difference in the costs of healthcare utilization between participants who received LoChro-Care and those who received usual care, regardless of whether the costs were evaluated 12 (adjusted mean difference € 130.99, 95%CI €-1477.73 to €1739.71, p = 0.873) or 18 (adjusted mean difference €192.99, 95%CI €-1894.66 to €2280.65, p = 0.856) months after the start of the intervention.

**Conclusion:**

This study revealed no differences in costs between older people receiving LoChro-Care or usual care. Before implementing the intervention, further studies with larger sample sizes are needed to provide robust evidence on the cost effects of LoChro-Care.

**Trial registration:**

German Clinical Trials Register (DRKS): DRKS00013904, https://drks.de/search/de/trial/DRKS00013904; date of first registration 02/02/2018.

**Supplementary Information:**

The online version contains supplementary material available at 10.1186/s12913-023-10401-1.

## Introduction

Against the background that older people often have chronic, mostly multiple illnesses and these are accompanied by physical, mental, and functional limitations, a new local, collaborative, stepped, and personalized form of care, the LoChro-Care intervention, was developed and evaluated [1–6]. LoChro-Care was designed to improve patients’ self-management in coordinating their individual care network [1, 7]. For this purpose, trained chronic care managers (CCM) provided assistance to establish contact to formal and informal support (e.g., general practitioner, family, regional geriatric outpatient services). In detail, LoChro-Care comprised (a) a comprehensive assessment of the patients’ health constitution and context, (b) the creation of a tailored healthcare plan that aligns with the patient’s prioritized healthcare issues and preferences, (c) the implementation, monitoring, and modification of the plan, and (d) a closing session [1, 7]. In the case of mild depression, diabetes, or the absence of a primary caregiver, extra interventional components were applied (problem solving therapy, skill training, trained volunteers). At least the first three contacts took place in the home environment, whereas the subsequent sessions could also be conducted by telephone. The intervention lasted 12 month, with 7–16 contacts with the CCM. As a result, patients’ health-related outcomes were expected to be improved or at least worsening progression delayed. Therefore, LoChro-Care was evaluated in terms of patients’ physical, psychological, and social health status (as indicated by functional health and depression), as well as their perceived heath care situation, health-related quality of life, life-satisfaction [7], and medication appropriateness.

The objective of the present study is to outline the effectiveness of LoChro-Care regarding the secondary endpoint of health resource utilization. Specifically, we hypothesized that LoChro-Care would lead to a more appropriate utilization of health and nursing care services in terms of decreased emergency hospitalizations, reduced non-elective hospital days and nursing home admissions, more adequate use of informal and formal community services, as well as enhanced disease self-management abilities that contribute to save health care costs [1].

## Methods

A two-group, parallel randomized controlled trial was conducted. Patients aged 65 + with one or multiple chronic conditions or geriatric symptoms (e.g., diabetes, hypertension, ischemic heart disease, atrial fibrillation) were recruited by research associates at inpatient and outpatient departments of the Medical Centre, University of Freiburg, Germany, between January 2018 and March 2020 [7]. Eligible patients were asked to participate in a short screening (“Identification of Seniors at Risk” questionnaire [8] to assess their risk of unplanned readmission and need for nursing care. Inclusion criteria required at least 2 positive responses out of 6 risk domains. Patients with terminal medical conditions and insufficient German language skills were excluded. Healthcare utilization costs are calculated by using an adapted version of the generic, self-reporting FIMA©- questionnaire [9–12] in combination with the application of standardized unit costs [13, 14]. Questionnaires were given at 3 time points (T_0_ baseline, T_1_ after 12 months, T_2_ after 18 months). Overall, utilization of 10 cost indicators (*General practitioner, Specialist, Day hospital, Hospitalization days [normal ward and intensive care days], Inpatient rehabilitation, Ambulatory nursing, Inpatient nursing, Remendies, Auxiliary means)* were measured and total healthcare utilization costs were calculated for a 3-month period prior to T_0_, T_1_ and T_2_. All costs are expressed in 2021 values and represent the perspective of the healthcare system.

Utilization of the different cost indicators at T_1_ and T_2_ was analyzed using negative binomial regression models with adjustment for age, sex and level of care (at baseline) as well as the utilization of the respective indicator at T_0_ [15]. In Germany, there are different levels of care, which also depends on the amount of financial support a patient receives from the statutory long term care insurance. To determine the level of care, an assessment is carried out, which evaluates the individual’s ability to perform everyday activities and the level of support required. As a higher level of care translates into more financial support from the compulsory long-term care insurance, the level of care may change frequently over time when the degree of care dependency increases.

In addition, joint analysis of T_1_ and T_2_ utilizations are applied using confounder adjusted negative binomial regression models with the patients ID as a random intercept to account for multiple records on the patient level. The results are shown as adjusted incidence rate ratios.

The overall 3-month costs of care at T_1_ and T_2_ are analyzed using generalized linear models with log-link and gamma distribution [16, 17]. Again, adjustment for age, sex, the level of care at 3-month costs prior to T_0_ took place. Joint analysis of 3-month costs at T_1_ and T_2_ were conducted using a population –averaged panel data model (with log-link and gamma distribution) to account for multiple records on the patient level. In a last step, the impact of baseline characteristics on overall 3-month costs of care were analyzed across all three periods (T_0_, T_1_ and T_2_). Furthermore, a population–averaged panel data model (with log-link and gamma distribution) was used to account for multiple records on the patient level. Included confounders were group, age sex and level of care. All analyses were performed using Stata 17 (Stata Corp., Texas, USA).

## Results

Three hundred thirty patients were eligible for the final investigation, which were well balanced between the groups (163 patients from the intervention group and 167 patients from the control group). Out of the 167 control group patients, 40.12% were males while in the intervention group, 46.01% were males. The mean age of the participants was 77.36 ± 6.60 and 76.19 ± 6.12 in the control group and intervention group, respectively. As shown in Table 1, most of the patients (78.44% and 76.07% in the control group and intervention group, respectively) were not eligible for long-term care benefits from the statutory long-term care insurance (level of care 0). With respect to the other levels of care, percentages are balanced between the groups.

**Table 1:** Baseline characteristics

		Control group (N = 167)		Intervention group (N = 163)	
Age	Mean, SD	77.36	6.60	76.19	6.12
Male sex	%	40.12%		46.01%	
Level of care 0	%	78.44%		76.07%	
Level of care 1		6.59%		6.13%	
Level of care 2		10.78%		11.66%	
Level of care 3		2.40%		5.52%	
Level of care 4		1.80%		0.61%	

When comparing the various cost indicators, no significant difference was found between the two groups. The total costs of health care utilization were at comparable levels in T_1_ (intervention group: M = 6656.79€, SD = 10709.03€; control group: M = 6178.09€, SD = 10595.24€) and T_2_ (intervention group: M = 6809.13€, SD = 9907.18€; control group: M = 6221.26€, SD = 9616.46€). The same is true for the 10 cost indicators that were collected for 3-month periods prior to T_0_, T_1_ and T_2_ (see Table 2).

**Table 2:** Healthcare utilization

**Within 3 months prior to T**_**0**_			**Control group** **(N = 167)**		**Intervention group (N = 163)**	
General practitioner	visits	Mean, SD	3.37	3.60	3.81	3.48
Specialist	visits	Mean, SD	5.25	5.20	6.01	6.55
Day hospital	visits	Mean, SD	0.54	1.19	1.09	2.96
Hospitalization, normal ward	days	Mean, SD	2.87	7.30	2.4	4.39
Hospitalization, intensive care	days	Mean, SD	0.22	1.00	0.12	0.4
Inpatient rehab	days	Mean, SD	0.97	3.16	1.92	5.48
Ambulatory nursing	hours	Mean, SD	73.65	280.72	101.74	314.1
Inpatient nursing	days	Mean, SD	0.27	2.46	1.12	7.88
Remendies	hours	Mean, SD	7.01	8.92	10.41	12.98
Auxiliary means	days	Mean, SD	3.84	1.94	3.9	1.88
overall 3-month costs	€	Mean, SD	6127.65	9603.77	7268.48	10793.35
**Within 3 months prior to T**_**1**_			**Control group** **(N = 167)**		**Intervention group (N = 163)**	
General practitioner	visits	Mean, SD	2.59	2.18	2.79	2.42
Specialist	visits	Mean, SD	4.22	6.30	4.16	3.59
Day hospital	visits	Mean, SD	0.82	1.99	0.72	1.31
Hospitalization, normal ward	days	Mean, SD	1.59	4.36	1.15	3.09
Hospitalization, intensive care	days	Mean, SD	0.08	0.42	0.02	0.23
Inpatient rehab	days	Mean, SD	1.48	4.33	1.58	4.49
Ambulatory nursing	hours	Mean, SD	95.35	292.32	119.85	318.99
Inpatient nursing	days	Mean, SD	0.56	3.93	0.24	1.58
Remendies	hours	Mean, SD	6.09	9.04	9.51	11
Auxiliary means	days	Mean, SD	4.38	1.98	4.39	1.91
overall 3-month costs	€	Mean, SD	6178.09	10595.24	6656.79	10709.03
**Within 3 months prior to T**_**2**_			**Control group** **(N = 158)**		**Intervention group (N = 150)**	
General practitioner	visits	Mean, SD	2.49	2.01	2.54	2.02
Specialist	visits	Mean, SD	3.78	5.40	3.72	3.71
Day hospital	visits	Mean, SD	0.83	2.69	0.94	3.02
Hospitalization, normal ward	days	Mean, SD	1.42	3.50	2.05	6.47
Hospitalization, intensive care	days	Mean, SD	0.11	0.86	0.14	0.67
Inpatient rehab	days	Mean, SD	1.01	3.35	1.19	4.07
Ambulatory nursing	hours	Mean, SD	102.08	254.63	99.2	267.28
Inpatient nursing	days	Mean, SD	0.45	5.49	0.26	2.02
Remendies	hours	Mean, SD	6.46	9.17	7.69	10.04
Auxiliary means	days	Mean, SD	4.55	1.94	4.53	2.05
overall 3-month costs	€	Mean, SD	6221.26	9616.46	6809.13	9907.18

Accordingly, no significant effect of group membership at T_1_ (adjusted mean difference € 130.99, 95%CI €-1477.73 to €1739.71, p = 0.873), T_2_ (adjusted mean difference €192.99, 95%CI €-1894.66 to €2280.65, p = 0.856) or over both measurement points together (adjusted mean difference €91.87, 95%CI €-1458.03 to €1641.77, p = 0.908) could be shown by regression analysis.

**Fig. 1:**
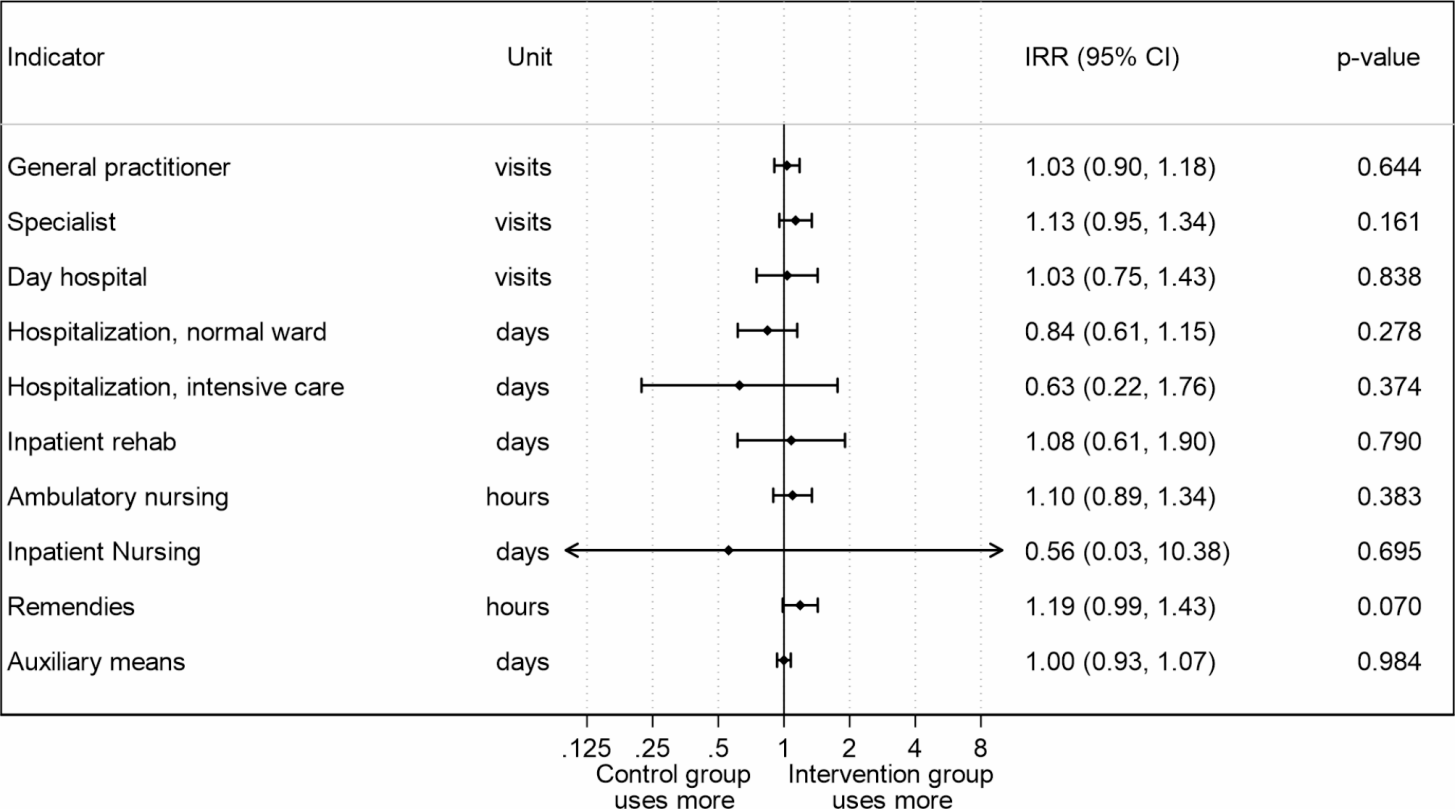
Analysis of cost indicators at T_1_ and T_2_

When analyzing different cost indicators at T_1_ and T_2,_ negative binomial regression models were performed. Figure 1 shows the corresponding incidence rate ratios when analyzing over both measurement points (T_1_ and T_2_). All cost indicators were statistically insignificant (p-values > 0.05). Similar results occur when analyzing T_1_ and T_2_ separately (see supplemental material, Figure S1 and S2). In summary, no statistically significant difference between the intervention group and the control group could be found in any of the endpoints.

**Fig. 2:**
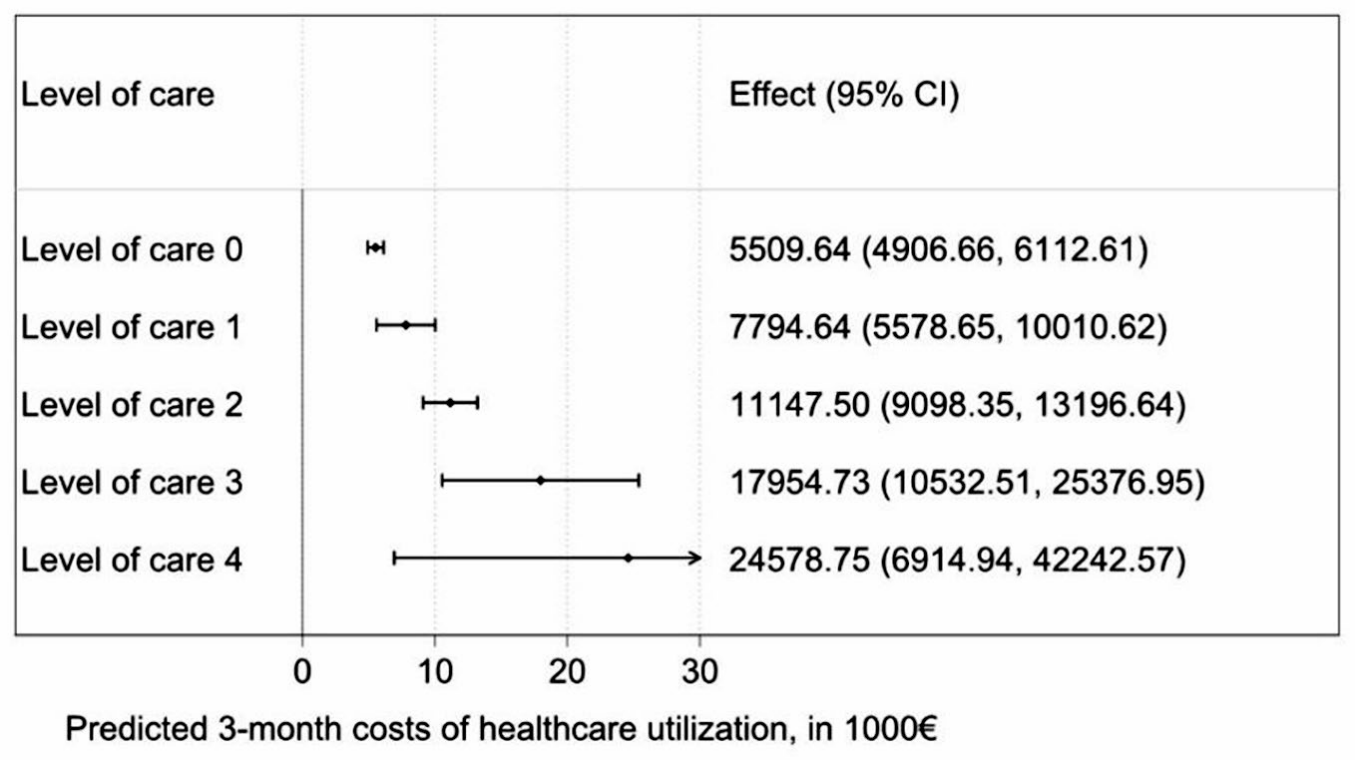
Analysis of potential confounders at T_0,_ T_1_ and T_2_

The analysis of potential confounders showed that group (Intervention vs. control group, p = 0.600), age (p = 0.499) and sex (p = 0.506) did not impact 3-month costs of care. The level of care, however, had a major impact on the 3-month costs of care. As shown in Fig. 2, a patient at care level 0 (N = 255) was associated €5509.64 costs of care (95%CI €4906.66 to €6112.61) while a patient at care level 2 (N = 37) was associated €11147.50 costs of care (95%CI €9098.35 to €13196.64).

## Discussion

The economic evaluation of our new local, collaborative, stepped, and personalized LoChro care management program showed no significant difference in health care costs between participants who received the LoChro-Care and those who received usual care, regardless of whether health care costs were measured 12 or 18 months after the start of the intervention. Thus, we did not find evidence to support our hypothesis that LoChro-Care would be associated with savings in health care costs. This result suggests that the overall extent of health care utilization progressed similarly between the two groups, regardless of the type of intervention they received.

In addition, our hypothesis - that LoChro-Care leads to more appropriate use of health and care services - was not supported. For the ten different cost indicators measured 12 and 18 months after the start of the intervention, we found no group differences. This means that participants who received LoChro-Care were not associated with the expected reduction in emergency hospital admissions, reduction in non-elective hospital days and nursing home admissions, more appropriate use of informal and formal community services, and improved ability to self-manage their condition, compared to participants who received usual care.

Our analysis however showed that 3-month health care costs of LoChro-Care are highly correlated with the patients’ formal level of care. In Germany, a structured assessment of care needs, e.g. for activities of daily living, mobility or personal hygiene, is used to determine the level of care and thus the amount of financial support a patient receives from the statutory long term care insurance. This financial support was not included in the healthcare utilization costs assessed in this study because it is provided by the German long term care insurance rather than the health care insurance, on which our analysis focused. In addition, because reducing the need for nursing care is a lengthy process, we hypothesized that LoChro-Care would offer potential for short-term health care cost savings rather than impacting long-term costs.

Direct comparison of the results with other studies offers a number of challenges. First, inclusion in the study occurred during a hospital contact. Secondly, inclusion in the study was based on a pre-assessment regarding the participants’ risk of unplanned readmission and need for nursing care. Nevertheless, the result of the LoChro study is in line with previous studies analyzing the costs of care among older patients in the outpatient sector [14, 18–23]. From the point of view of the intervention, Kari’s study appears to be the most suitable for direct comparison with the present results. Unfortunately, the site of inclusion differs substantially between the studies: in Kari’s study, patients were invited to participate by letter and irrespective of a hospital contact, whereas in the present study, patients were approached during a hospital contact combined with a pre-assessment of the severity of underlying conditions. Without going into detail about the intervention, Kari’s people-centered care model is quite comparable to the LoChro intervention. The same applies to the results regarding the impact of the intervention on the cost of care. Neither in the first year of the study (p = 0.31) nor in the second year (p = 0.76), nor over both years together (p = 0.42) a difference between intervention and control group could be shown in the study by Kari et al. [18]. A look at the individual components of the costs analysed by Kari and colleagues also showed no trend towards a more appropriate use of health and care services (less emergency admissions, hospital stays) between intervention and control group [18]. In contrast, intervention programs for multimorbid older people, which were found to be cost effective, were characterized by an earlier start in the development of chronic multimorbidity (e.g. with preventive home visits) [24], or comprised not only support for self-management but also active therapeutic measures such as home safety modifications [25, 26], or mobility training [27]. LoChro care adaptions in this direction might be reasonable, followed by a re-evaluation of the adapted program.

## Limitations

Taking into account that LoChro-Care was a novel intervention being implemented for the first time, some limitations should be mentioned. First, the self-reporting nature of the questionnaires may have resulted in recall biases, especially in face of our target sample of older people.

A substantial limitation regarding the external validity could be the regional specificity of the study. The conduct of the study was limited to the area of Freiburg and the surrounding area. The implementation of the intervention and the study results may have been influenced by specific characteristics of this area, such as relatively high socioeconomic performance. Moreover, we excluded patients with terminal illnesses and insufficient knowledge of German.

Although the sample size could be considered considerable in the context of geriatric research, it was relatively small in terms of a cost-effectiveness analysis. Given the enormous standard deviation in total costs (see Table 2 for details), a multiple of the current sample size would have been necessary to show even a moderately large difference in cost values. Moreover, we have limited ourselves to a simple cost-cost comparison from the perspective of the health care system. The background to this is the ineffectiveness of the LoChro trial regarding the endpoints of physical, psychological, and social health status as well as health-related quality of life and life satisfaction [7], as well as the lack of difference in service utilization between the groups. For the same reasons, the costs of the intervention were not calculated. However, even though this study has shown negative findings, the “Absence of evidence is not evidence of absence” [28].

## Conclusion

This study revealed no differences in costs between older people receiving our new local, collaborative, stepped, and personalized LoChro-Care management program or usual care. Keeping in mind the relatively small sample size per economic standards, there is currently no economic incentive for a wider implementation of the intervention. Further studies with larger sample sizes are needed to provide robust evidence of cost savings or cost neutrality of LoChro-Care.

### Electronic supplementary material

Below is the link to the electronic supplementary material.

**Supplementary Material 1: Figure 1:** Analysis of cost indicators at T_1_. **Figure 2:** Analysis of cost indicators at T_2_

## Data Availability

The datasets used and/or analysed during the current study available from the corresponding author on reasonable request.
