# Supplementary material for: The economic impact of a local, collaborative, stepped, and personalized care management for older people with chronic diseases: results from the randomized comparative effectiveness LoChro-trial
Source: BMC Health Serv Res. 2023 Dec 15;23:1422. doi: 10.1186/s12913-023-10401-1 (PMC10724907; doi:10.1186/s12913-023-10401-1)
Supplement: Supplementary file 1 — Supplementary Material 1: Figure 1: Analysis of cost indicators at T1. Figure 2: Analysis of cost indicators at T2 [file 12913_2023_10401_MOESM1_ESM.docx]

**Supplemental Figure 1: Analysis of cost indicators at T_1_**

**Supplemental Figure 2: Analysis of cost indicators at T_2_**
